# Supplementary material for: Programmable generation of counterrotating bicircular light pulses in the multi-terahertz frequency range
Source: Nat Commun. 2024 Jul 26;15:6310. doi: 10.1038/s41467-024-50186-3 (PMC11282245; doi:10.1038/s41467-024-50186-3)
Supplement: Supplementary file 1 — Supplementary Information [file 41467_2024_50186_MOESM1_ESM.pdf]

Supplementary Information

Programmable generation of counterrotating bicircular light pulses in the multi-terahertz frequency range

Kotaro Ogawa[1], Natsuki Kanda[1,2]*, Yuta Murotani[1], and Ryusuke Matsunaga[1]*

[1]*The Institute for Solid State Physics, The University of Tokyo, Kashiwa, Chiba 277-8581, Japan*

[2] *Ultrafast Coherent Soft X-ray Photonics Research Team, RIKEN Center for Advanced Photonics, RIKEN, 2-1 Hirosawa, Wako, Saitama 351-0198, Japan*

*e-mail: n-kanda@riken.jp, matsunaga@issp.u-tokyo.ac.jp

<u>Supplementary Note 1:</u> Transients of BCL pulses with a long scan range

As explained in the main text, a key idea in this work is to control the unwanted near-infrared (NIR) bands (gray area in Fig. 1d) to be co-circularly polarized with additional 400-fs retardation from the main pulse, which prohibits the difference frequency generation (DFG) from the unwanted NIR bands because of the angular momentum selection rule. To confirm this, we measured the electric field trajectories of bicircular light (BCL) pulses with a scan range as long as 1 ps. Figure S1a shows the *x*- and *y*-axis electric field trajectories of a BCL pulse composed of the central frequencies of 17 and 34 THz, and Fig. S1b shows the three-dimensional (3D) plots of the waveform. The black arrow in Fig. S1a indicates the time with a 400-fs retardation from the main pulse. Around this region, multi-terahertz field does not perfectly vanish. The DFG from the unwanted NIR bands might be included here. However, free induction decay of absorption lines of CO_2 and H_2O molecules at around 25 and 40 THz, respectively, and the backside reflection inside the 10 µm-thick electro-optic (EO) crystal would also appear in this time region. Compared with the main pulse, the delayed multi-terahertz electric field including these contributions are sufficiently weak (see Supplementary Information Note 8) so that they do not pose an obstacle to apply the main pulse to control physical properties of matter.

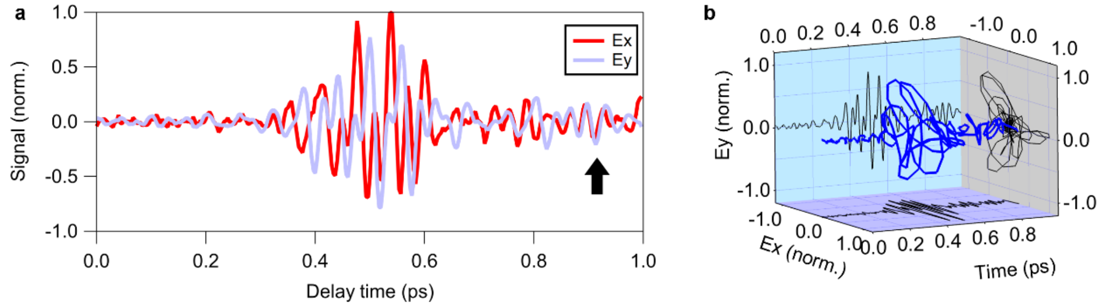

Fig. S1. The BCL trajectory measured with a time window of 1 ps. a, Multi-terahertz BCL pulse trajectory along *x*-axis (red line) and *y*-axis (light blue line). **b**, A 3D plot of the field trajectory.

<u>Supplementary Note 2:</u> Relation between the relative phase and the orientation of trajectory

Here we derive the formula relating the orientation angle $\omega_R \alpha/(\omega_L + \omega_R)$ of the BCL

trajectory to the relative phase α between the left-circularly polarized (LCP) and right-circularly polarized (RCP) electric fields. We simplify the electric field amplitude for each color as a time-independent constant: $E_L(t) = E_L$ and $E_R(t) = E_R$ in Eq. (2). Then, a BCL electric field can be expressed as

$$\boldsymbol{E}(t) = \mathrm{Re}\left(E_L \boldsymbol{e}_L e^{i(\omega_L t+\alpha)} + E_R \boldsymbol{e}_R e^{i\omega_R t}\right). \tag{S2.1}$$

Considering only the real part and moving to the linearly polarized bases, we obtain

$$\boldsymbol{E}(t) = \frac{1}{\sqrt{2}}\begin{pmatrix} E_L\cos(\omega_L t+\alpha) + E_R\cos(\omega_R t) \\ E_L\sin(\omega_L t+\alpha) - E_R\sin(\omega_R t) \end{pmatrix}, \tag{S2.2}$$

$$|\boldsymbol{E}(t)|^2 = \frac{1}{2}\{E_L^2 + E_R^2 + 2E_L E_R\cos[(\omega_L+\omega_R)t+\alpha]\}. \tag{S2.3}$$

Equation (S2.3) takes the maximum when $\cos[(\omega_L+\omega_R)t+\alpha] = 1$, *i.e.*, $t = (2m\pi - \alpha)/(\omega_L+\omega_R)$, where m is an integer. Each of these times corresponds to an apex in the field trajectory. We define these times as $t_{\max}$ and substitute it into Eq. (S2.2), to obtain

$$\boldsymbol{E}(t_{\max}) = \frac{E_L+E_R}{\sqrt{2}} R\left(\frac{\omega_R}{\omega_L+\omega_R}\alpha\right) R\left(\frac{\omega_L}{\omega_L+\omega_R}2m\pi\right)\begin{pmatrix}1\\0\end{pmatrix}, \tag{S2.4}$$

where $R(\theta)$ is the rotation matrix by angle θ. Thus, the BCL trajectory rotates by $\omega_R\alpha/(\omega_L+\omega_R)$ if the relative phase α is given to the LCP pulse.

The same calculation can be performed when the relative phase α_R is given to the RCP light, and the rotational angle $-\omega_L\alpha_R/(\omega_L+\omega_R)$ is obtained. Therefore, if the relative phase is given to the *L*-band instead of the *U*-band, the electric field trajectory will rotate according to the latter formula. As an example, we consider a BCL pulse with $\omega_L = 2\omega_R$. When a relative phase $\alpha_L = \pi$ rad is given to ω_L, the trajectory rotates by $\pi/3$ rad. On the other hand, when a relative phase $\alpha_R = \pi$ rad is given to ω_R, the BCL pulse gets the orientation of $-2\pi/3$ rad.

Supplementary Note 3: Orientation control of BCL pulses with other symmetries

Because the BCL orientation can be controlled by the relative phase between the NIR *U*- and *L*-bands, it is applicable to all the BCL pulses generated by this proposed method. In addition to the trefoil-like patterns in Figs. 1a.2 and 1a.3, we plot the change in the orientation of the triangle-like C_3 BCL trajectory in Figs. S2a-d. We added offset phases of 0.2π, 0.7π, 1.2π, and 1.7π to *U*-band, corresponding to the orientation of 0°, 30°, 60°, and 90°. These setting angles are indicated by the red dotted lines in Figs. S2a–d, respectively. Furthermore, Figs. S2e–h show the change in the rotational orientation of the star-like C_5 BCL trajectory. We added offset phases of 0, 0.5π, 1.0π, and 1.5π,

corresponding to the orientation of 0°, 36°, 72°, and 108°, respectively. These setting angles are also indicated by the red dotted lines in Fig. S2e–h. These results show that the orientation control is possible for any trajectories.

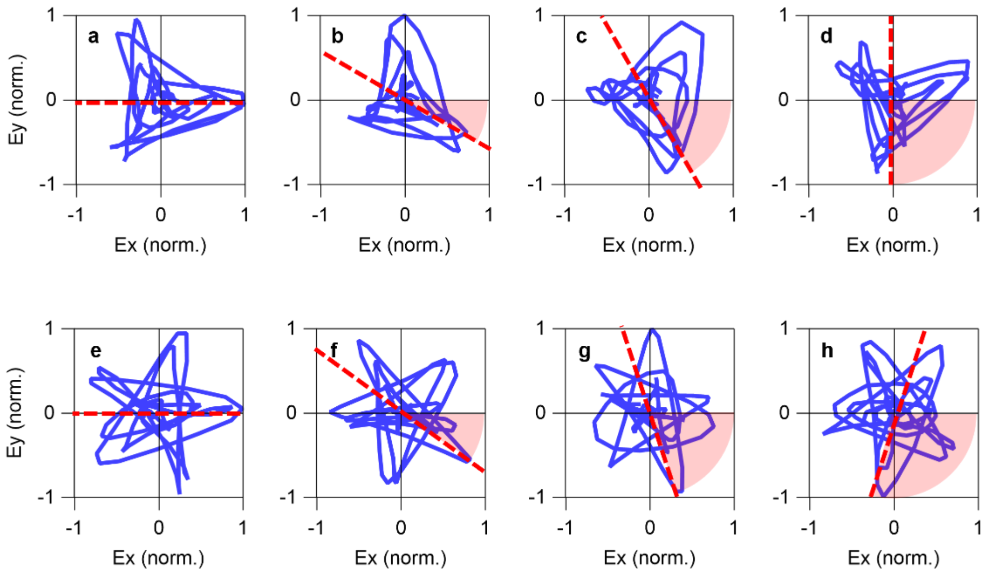

Fig. S2. Control of the orientation of BCL pulses with C_3 and C_5 symmetries. a–d, the orientation control of triangle-like BCL trajectory. **e–h,** the orientation control of star-like BCL trajectory.

<u>Supplementary Note 4:</u> Power spectra and 3D plots corresponding to the data in Fig. 3

In Fig. 3, we demonstrated the control of rotational symmetry by modulation of the frequency ratio. Here, the intensity spectra and 3D plots of electric field trajectories for each case are shown in Fig. S3. The two-color frequencies described in Fig. 3 (*e.g.*, 17 THz + 34 THz in Fig. 3a) are the peak values of each intensity spectrum. For each frequency ratio, the intensity ratio was adjusted to clearly show the rotational symmetry, and the relative phase was adjusted so that the apex of one arm extends in the *x*-axis direction for visibility.

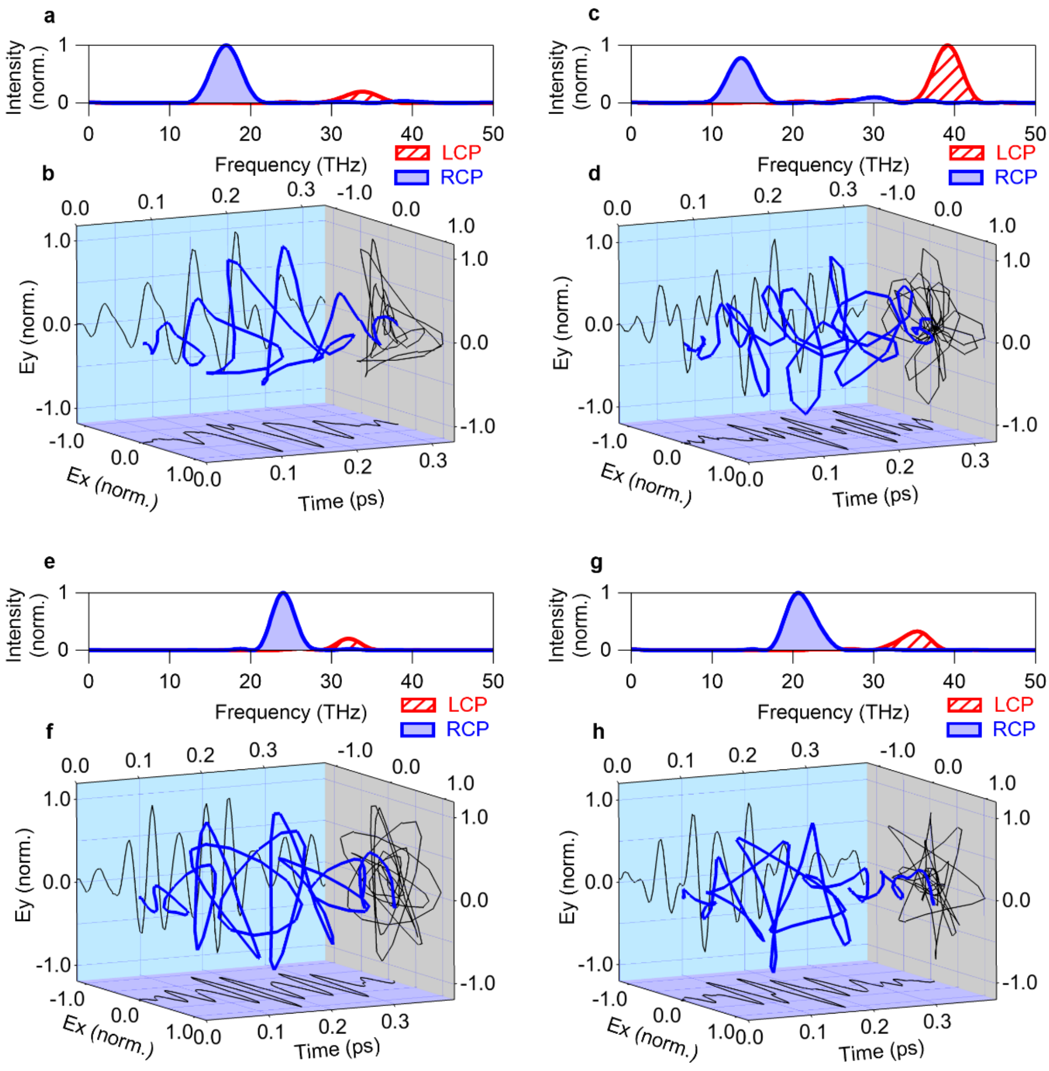

Fig. S3. The intensity spectra and 3D plots of BCL pulses with controlled rotational symmetry. Each trajectory has (**a** and **b**) C_3 in Fig. 3a, (**c** and **d**) C_4 in Fig. 3b, (**e** and **f**) C_7 in Fig. 3c, or (**g** and **h**) C_8 in Fig. 3d symmetry.

Supplementary Note 5: FROG measurement of compressed NIR pulse

We describe the evaluation of compressed NIR pulses by the frequency-resolved optical gating (FROG) method[S1] in detail. The measured and retrieved FROG traces using second harmonic generation (SHG) are shown in Fig. S4a, which were measured after the multi-plate broadening and compression.[S2] The group delay dispersion (GDD) is overcompensated by chirp mirrors considering the additional dispersion in POMEOS setup. For the pulse evaluation, we inserted three fused-silica plates with thickness of 12,

5, and 1 mm to realise the shortest pulse. The FROG error was 1.7% over 256×256 retrieval grid. Figure S4b shows the retrieved intensity and phase spectra. Figure S4c illustrates the time-domain waveform of the intensity, showing that the NIR pulse was well compressed although a certain degree of pedestal is discerned. The retrieved pulse width of the NIR compressed pulse at full width at half-maximum was measured as 11.9 fs. The Fourier-transform limit pulse is also shown in Fig. S4c, whose duration is 11.7 fs, indicated by the black arrows. The sampling light was dispersion compensated by 1 mm-thick fused-silica plates before POMEOS to make it as close as possible to the Fourier-transform limit pulse when injected into the EO crystal, considering the dispersions in the transmission components in POMEOS (lens, waveplate, and PEM).

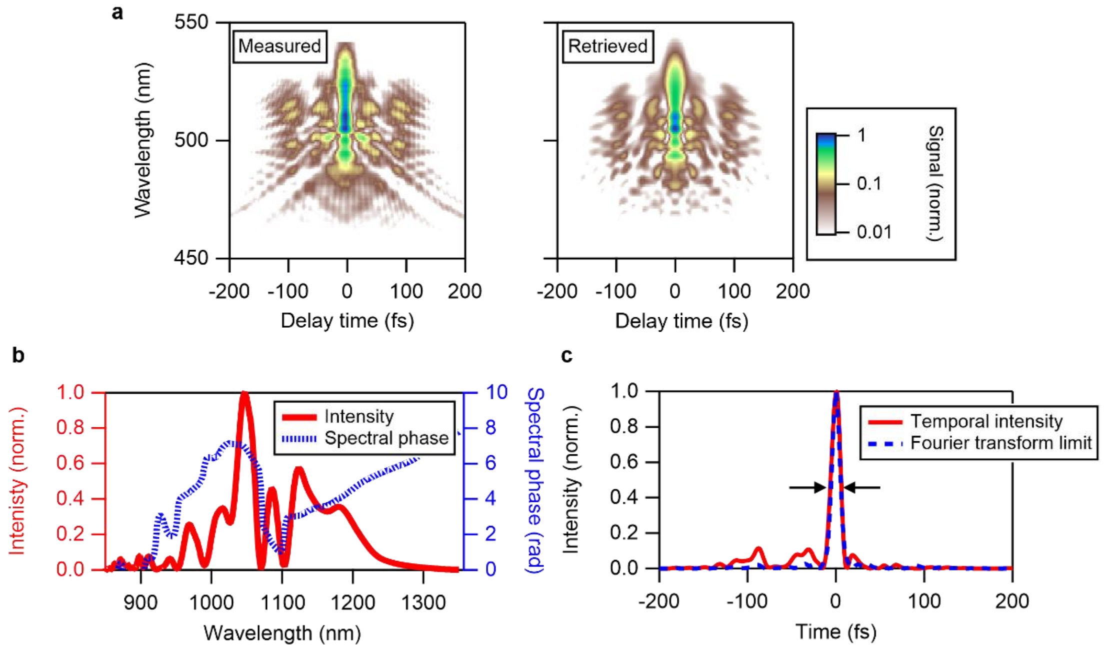

Fig. S4. FROG measurement of NIR compressed pulse. a, Measured and retrieved SHG-FROG traces after the output of the multi-plate pulse broadening. **b**, Retrieved intensity spectrum (red) and spectral phase (blue). **c**, Time-domain retrieved intensity envelope (red) and its Fourier transform limit pulse (blue). The black arrows show the FWHM.

<u>Supplementary Note 6:</u> Relative phase stability of BCL pulses

Here, we describe the relative phase stability in detail. To evaluate it, we performed 1-hour measurements of BCL electric field trajectories with the polarization-modulated

electro-optic sampling (POMEOS[S3]), each of which consisted of 100-point measurements over a 50 µm scan range (0.33 ps in delay time range) for each polarization direction, taking about 1 min for a scanning. For each delay point, 900 laser shots (300 ms) are accumulated. Such measurements were made continuously by 51 times in 1 h. Figures S5a, b and d illustrate the absolute values of the electric field trajectory $E_{\mathrm{abs}} = \sqrt{E_x^2 + E_y^2}$ (**a**), the electric field trajectory in the *xy* plane (**b**), and the power spectra of LCP and RCP components (**d**) for a single typical measurement, showing that the measured BCL pulses are centred at frequencies of 20 and 30 THz.

The fluctuations of the BCL electric field trajectory can be classified into two categories: (i) fluctuation in the axis of arrival time, which can be called a timing jitter, and (ii) in-plane (*xy*) fluctuation.

(i) The time jitter can be evaluated by the fluctuation of the peak position of the absolute value of the electric field trajectory, E_{abs}, in the time direction (fluctuation in the direction of the black arrow on the upper part of Fig. S5a). The peak position fluctuation in time originates from two sources: (a) a jitter between the sampling light and the envelope of BCL pulse and (b) fluctuation of the carrier envelope offset phase of the BCL pulse itself. But it is very difficult to separate the two due to the characteristic of BCL as a pulse with two colors mixed together. Since both of these jitters should be kept as small as possible for future applications, we combined the two origins and evaluated it as a single parameter of the peak position fluctuations in time. The lower part of Fig. S5a shows the absolute value of the electric field amplitude E_{abs} measured in 1 hour, which shows that there is little jitter or shift in the time direction for each pulse over a long period of time. The standard deviation of the absolute peak time was estimated to be as small as 0.23 fs.

(ii) Fluctuations of the electric field trajectory in the *xy* plane can be decomposed into amplitude and rotation directions. In this work, we evaluate the rotation direction, which is more important for the application to the control of physical properties (fluctuations in the direction of the black arrow in Fig. S5b). Fluctuations in the rotational direction originate from fluctuations in the relative phase of the two colors. For example, if there is a shift of relative phase $\Delta\alpha_L$ for the LCP components of frequency ω_L and $\Delta\alpha_R$ for the RCP components of frequency ω_R, the rotation angles derived from them can be calculated as

$$\begin{cases} \Delta\theta_L = \dfrac{\omega_R \Delta\alpha_L}{\omega_L + \omega_R} \\ \Delta\theta_R = -\dfrac{\omega_L \Delta\alpha_R}{\omega_L + \omega_R} \end{cases}, \tag{S6.1}$$

respectively (see Supplementary Information Note 2), and the sum of them equals the rotational angle. Figure S5c shows the instantaneous azimuthal angles of the electric field over a 1-hour measurement. We evaluated the instantaneous azimuthal angle change at one apex of an arm as the stability of the rotation angle of the trajectory . The standard deviation of the instantaneous azimuthal angle at the apex where the electric field intensity in the *x*-axis direction reaches its maximum was estimated to be 14.7 mrad.

From the acquired electric field trajectories, the CEP fluctuations of the 20 and 30 THz-centered components were analyzed. The electric field trajectory was decomposed into LCP and RCP components and their respective carrier-envelop phases (CEPs) were obtained. From this CEP shift of each component, rotational angles can be calculated using Eq. S6.1. Figure S5e shows the rotational angle $\Delta\theta_L$ (red square points) and the rotational angle $\Delta\theta_R$ (blue circle points). Then the sum of the rotational angles $\Delta\theta_L + \Delta\theta_R$ was calculated (green triangle points).

To examine the origin of the shift of rotational angles $\Delta\theta_L$ and $\Delta\theta_R$, let us consider differences of the optical path length in the 4f setup. We define the fluctuation of the optical path length as ΔL. The phase fluctuation are $(\Omega_0 + \omega_L)\Delta L/c$ (*U*-band), $\Omega_0 \Delta L/c$ (*C*-band), and $(\Omega_0 - \omega_R)\Delta L/c$ (*L*-band), respectively, where c is speed of light. Then, the phase fluctuations in DFG are expressed as

$$\begin{cases} \Delta\alpha_L = \dfrac{\omega_L \Delta L}{c} \\ \Delta\alpha_R = \dfrac{\omega_R \Delta L}{c} \end{cases}. \tag{S6.2}$$

By substituting to Eqs. S6.1 and S6.2, the rotation angle fluctuations for LCP and RCP components $\Delta\theta_L$ and $\Delta\theta_R$ are

$$\Delta\theta_L = -\Delta\theta_R = \frac{\omega_R \omega_L}{\omega_L + \omega_R}\frac{\Delta L}{c}. \tag{S6.3}$$

Here, the main cause of optical path length difference change in the 4f system might be thermal expansion due to temperature change of the system: the 4f optical system was constructed on an optical breadboard made by aluminum, and the change in thermal expansion coefficient of the breadboard due to temperature change produces the optical

path length change in the $4f$ optical system. If we define the temperature change as $\Delta\Theta$, the optical path length change is described as $\Delta L = \xi_{\mathrm{Al}} L_0 \Delta\Theta$, where $\xi_{\mathrm{Al}} = 23$ μm/(m·K) is the coefficient of aluminum thermal expansion and L_0 =0.6 m is the path length of the $4f$ optical system. Then, the contribution of temperature change for the rotation angle can be estimated as $\Delta\theta_L/\Delta\Theta = -\Delta\theta_R/\Delta\Theta = 3.5\ \mathrm{rad/°C}$. From the 1-h measurement results (Fig. S5e), $\Delta\theta_L$ is the order of 100 mrad which corresponds to the temperature change of 0.03 °C. To examine the consistency, we measured the temperature change inside the box of $4f$ system by a temperature data logger (TR72A, T&D), and confirmed that the change is less than 0.1℃.

Note that this common pass length change ΔL induces the rotation angles change $\Delta\theta_L$ and $\Delta\theta_R$, but the total orientation angle change $\Delta\theta = \Delta\theta_L + \Delta\theta_R$ is completely cancelled. This behavior is also consistent with our experimental results. The residual fluctuation might be caused by other reasons such as fluctuation of air, mechanical vibration, the thermal index changes of liquid crystal in SLM.

In our case, our passive stabilization scheme was successful for realizing highly stable orientation angle of the BCL trajectory. We also attempted to stabilize the temperature of the 4f system by attaching a heatsink with a temperature-stable chiller to the breadboard, the stability becomes worse than without chiller. That might be because of the vibration by the water flow for a chiller.

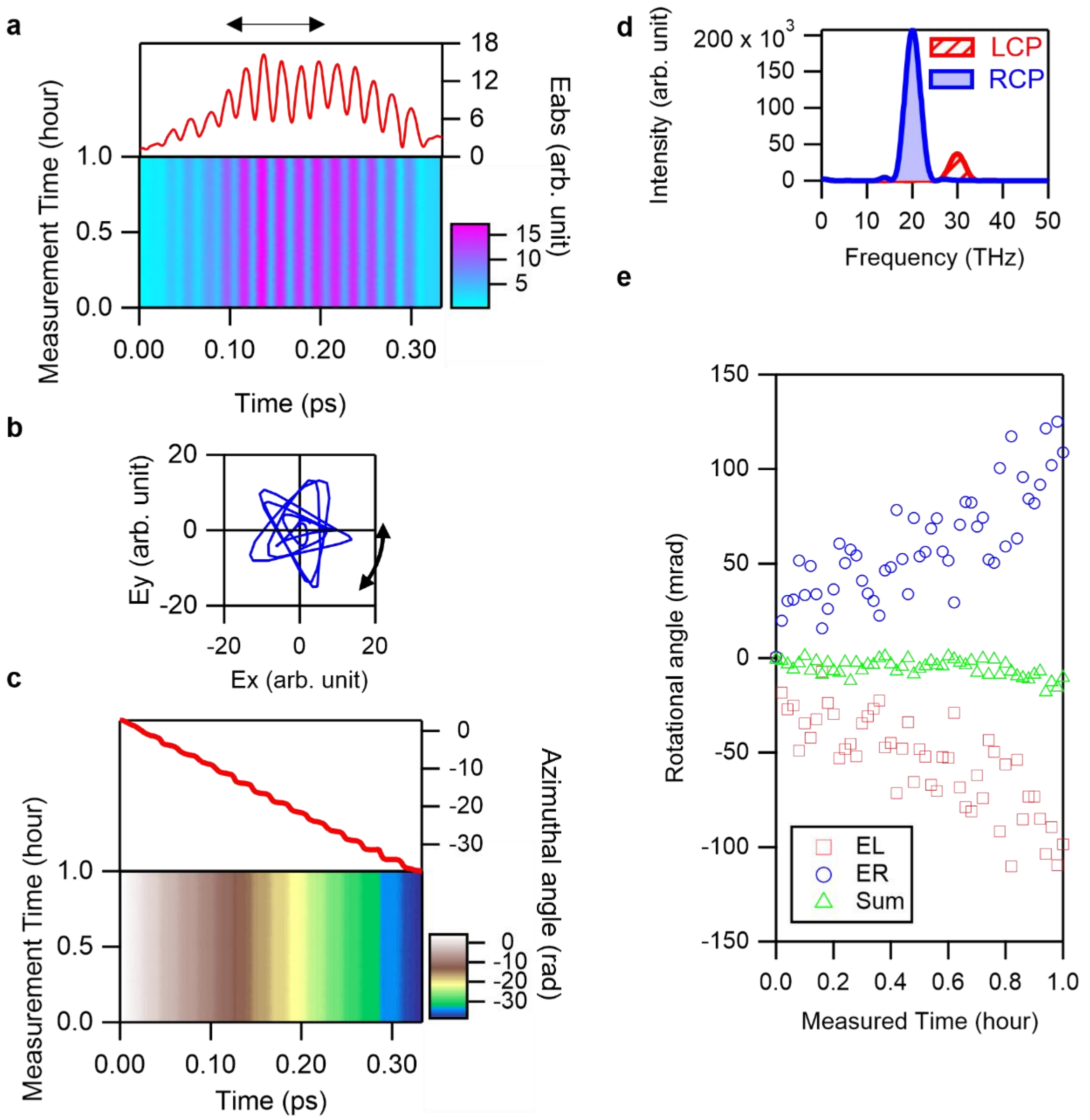

Fig. S5. Phase stability of BCL pulses. a, Time-dependent fluctuation of the absolute electric field amplitude of BCL pulses (lower) and its single typical measurement (upper). The black arrow shows the direction of the time-dependent fluctuation. **b**, Single typical measurement of the trajectory in the *xy*-plane. The black arrow shows the direction of the rotational-angle fluctuation. **c**, Azimuthal angles in time-domain (lower) and its typical measurement (upper). **d**, Power spectra of LCP and RCP components of the single typical measurement. **e**, Rotational angle change over 1h measurement.

<u>Supplementary Note 7:</u> Estimation of BCL electric field amplitude

This section details our estimation of the BCL electric field amplitude. First, we measured

the electric field amplitude of a simple linearly polarized multi-terahertz pulse as follows. Afterwards the BCL electric field amplitude was evaluated using the proportionality between the POMEOS signal and the electric field.

The pulse energy of the linearly polarized multi-terahertz pulse was estimated as about 0.3 nJ by a power meter (3A-P-THz, Ophir); the beam diameter was found to be 45 μm by a mid-infrared camera (RIGI-S2, Swiss Terahertz); and the pulse width was measured as 19 fs by considering FWHM obtained from Gaussian fitting to the EO sampling signal. From the above, the electric field amplitude of the linearly polarized multi-terahertz pulse was calculated to be about 700 kV/cm. From a comparison of the signal intensity, the electric field amplitude at the peak of our typical trefoil BCL pulse (shown in Fig. 2a) was estimated to be 100 kV/cm. The peak electric fields of the other BCL pulses were only a few times different from the trefoil BCL pulse.

Supplementary Note 8: Quality of the generated BCL pulses

Here, we characterize the quality of generated BCL pulses in time domain. We analyzed the trefoil-like BCL pulse shown in Fig. 2a, taken as a typical example. It is difficult to fit a BCL time-domain waveforms directly to a Gaussian function due to mixing of two colors. Since the two colors belong to the opposite helicity, they can be distinguished by moving to the circularly polarized bases. Therefore, we applied inverse Fourier transform to the LCP and RCP spectra calculated in the frequency space, so that we obtained the corresponding waveforms in time domain. The LCP (RCP) component is shown in Fig. S6a (S6b), along with the envelope obtained by Hilbert transform. The envelopes of the electric field trajectories of these LCP (RCP) components were Gaussian-fitted as illustrated in Fig. S6c. The FWHMs of the intensity envelopes were estimated as $\sigma_L = 0.21$ ps and $\sigma_R = 0.24$ ps, respectively. From the fitting, we also obtained the times t_L and t_R at which the Gaussian fits to the LCP and RCP components reach the maximum, respectively.

Two parameters characterize how close the generated BCL pulse is to the ideal one: (i) pulse overlap and (ii) the difference in pulse widths between the two colors. (i) Pulse overlap can be evaluated by $\tau = (t_L - t_R)/(\sigma_L + \sigma_R)$. We obtain $\tau = 0.032$ in the situation shown in Fig. S6c. (ii) The difference in pulse widths between the two colors can be defined as $\delta = (\sigma_L - \sigma_R)/(\sigma_L + \sigma_R)$. We find $\delta = -0.070$ in the situation shown in Fig. S6c. Ideally, these two indicators become to 0, and especially pulse overlap is consistent with good accuracy.

Another measure of the pulse quality is given by the pulse contrast, *i.e.*, the ratio of the intensity I_p at the peak to the intensity I_n of the noise. In Fig. S1, this ratio was calculated to be $I_p/I_n = 26$, showing a good pulse contrast.

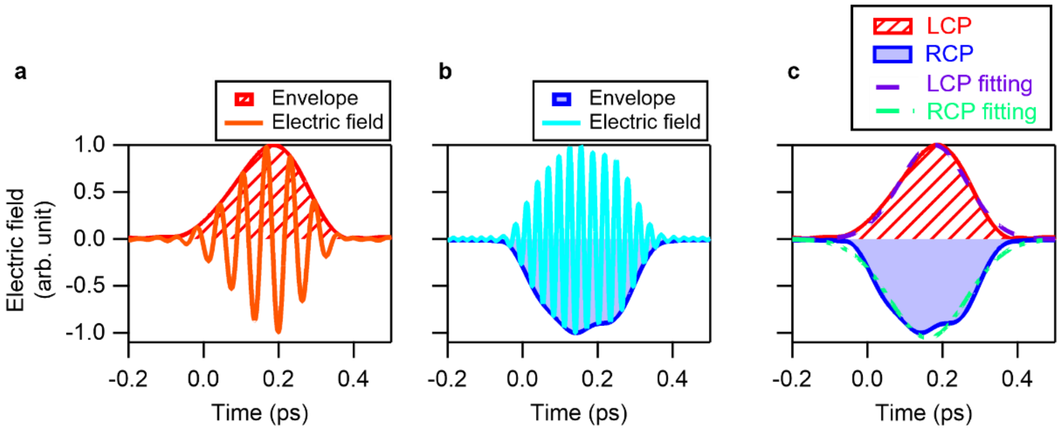

Fig. S6. A trefoil BCL pulse trajectory of LCP and RCP components. a, The x component of the electric field and its envelope of the LCP component. **b**, The x component of the electric field and its envelope of the RCP component. **c**, The envelope and its Gaussian fitting of LCP and RCP components.

Supplementary References

S1. Trebino, R. Frequency-resolved optical gating: the measurement of ultrashort laser pulses. *Springer Science & Business Media*, (2012).

S2. Kanda, N., Ishii, N., Itatani, J. & Matsunaga, R. Optical parametric amplification of phase-stable terahertz-to-mid-infrared pulses studied in the time domain. *Opt. Express* **29**, 3479-3489 (2021). 10.1364/OE.413200, Pubmed:33770945.

S3. Kanda, N., Nakagawa, M., Murotani, Y., & Matsunaga, R. Time-domain characterization of electric field vector in multi-terahertz pulses using polarization-modulated electro-optic sampling. *Opt. Express* **32**, 1576-1584 (2024). 10.1364/OE.506405
